# Research Advances on the Functions of Transcriptional Regulators in Filamentous Fungi

**DOI:** 10.3390/microorganisms14091892

**Published:** 2026-08-26

**Authors:** Jia Su, Xugang Lin, Yina Xia, Jing Zhao, Yue Chen, Depei Wang, Xianli Xue

**Affiliations:** 1 Key Laboratory of Industrial Microbiology & Engineering Research Center of Food Biotechnology of Ministry of Education, College of Biotechnology, Tianjin University of Science and Technology, Tianjin 300457, China; sujia4015@mail.tust.edu.cn (J.S.); lxg5024@mail.tust.edu.cn (X.L.); xyn@mail.tust.edu.cn (Y.X.); yuechen@tust.edu.cn (Y.C.); 2 COFCO Nutrition and Health Research Institute, Beijing 102209, China; m18819469616@163.com; 3 Key Laboratory of Industrial Fermentation Microbiology, Ministry of Education, Tianjin University of Science and Technology, Tianjin 300457, China

**Keywords:** filamentous fungi, transcription factors, genetic modification, regulatory functions, growth and development

## Abstract

Filamentous fungi are ubiquitous eukaryotic microorganisms characterized by multicellular architectures and branched hyphal structures. They are used in industrial production for the manufacture of enzyme preparations, various organic acids, and secondary metabolites. Transcription factors serve as core regulatory molecules governing vital biological processes in filamentous fungi. This review systematically elaborates on the classification, structural features, and functional diversity of transcription factors in filamentous fungi, with a focus on their regulatory roles in hyphal growth, morphological differentiation, metabolite synthesis, and environmental stress responses. We further summarize the key transcription factors in three representative fungal genera, namely, *Aspergillus*, *Trichoderma*, and *Penicillium*. Additionally, the potential applications of genetically engineering transcription factors to increase the yield of metabolites are discussed. This review provides a comprehensive theoretical framework for the genetic modification and high-value utilization of filamentous fungi.

## 1. Transcription Factors

### 1.1. Transcriptional Regulation Mechanisms

Transcription factors (TFs) are functional proteins that specifically recognize and bind to conserved DNA motifs. By binding to promoters or enhancers located upstream of target genes, they ultimately initiate, terminate, enhance, or attenuate the transcription of regulated genes [1].

Transcription factors recognize DNA in a combinatorial and synergistic manner. Through the integration of intracellular and extracellular signals, and under the regulation of transcription factor expression, localization, post-translational modifications, and chromatin remodeling, they recruit the transcriptional machinery to precisely regulate the expression of multiple genes [2]. Transcriptional regulators are classified into global transcriptional regulators and specific (local) transcriptional regulators. Among them, global transcriptional regulators such as LaeA or VeA are responsible for regulating hundreds of genes in cells; participate in global physiological processes, including cellular basal metabolism, stress response, and growth; or regulate multiple sets of functionally unrelated operons [3,4]. Conversely, specific transcriptional regulators such as NcSR or AtrR only regulate a small number of functionally related genes or operons.

**Figure 1:**
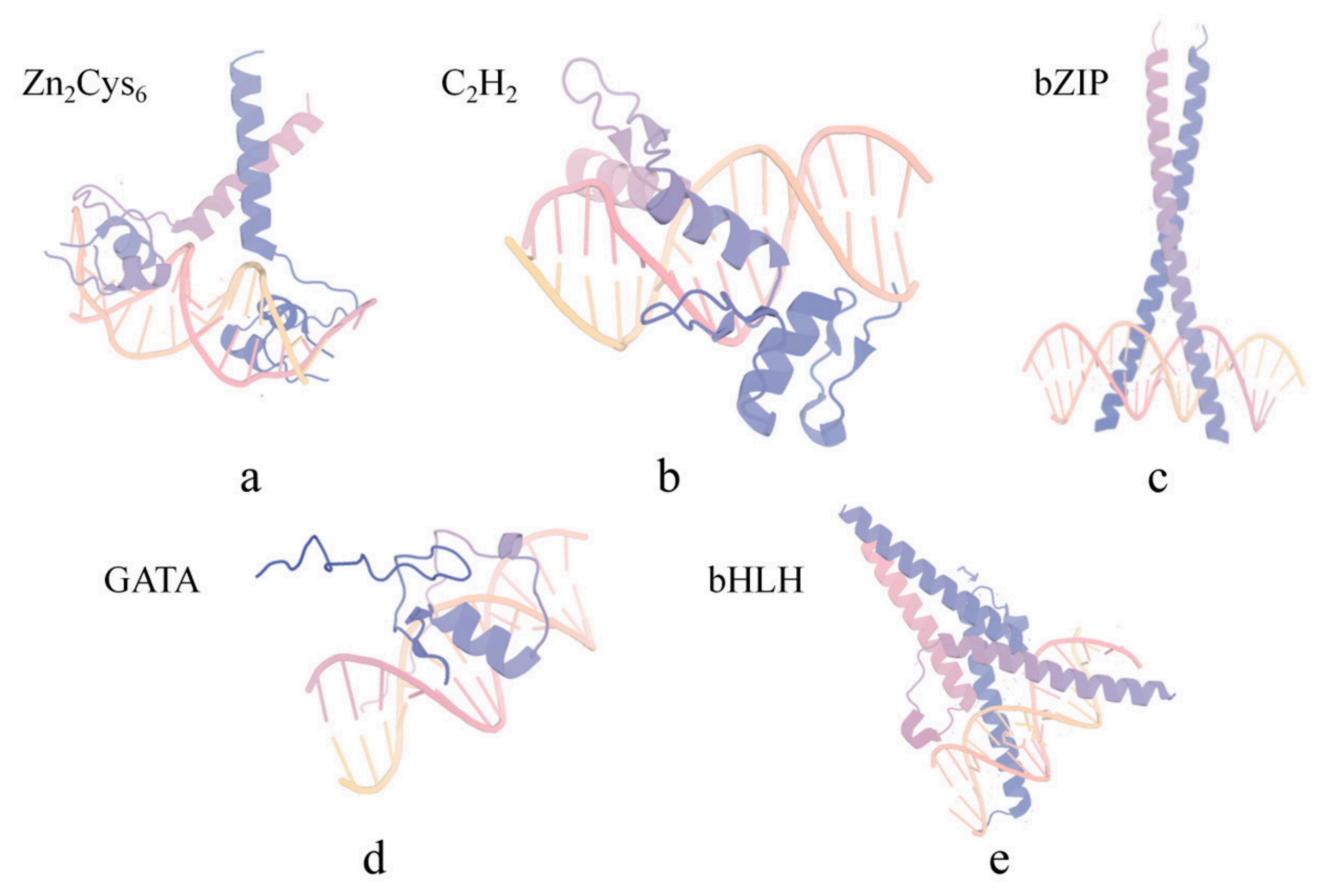
Transcription factor classification in filamentous fungi. The structural models were retrieved from the RCSB Protein Data Bank (RCSB PDB).

In general, transcription factors contain multiple independent functional domains, including the DNA-binding domain (DBD) and transcription regulatory domain (TRD), which are responsible for recognizing and binding to specific DNA sequences and regulating transcription, respectively [5]. Common types of DBDs include the zinc cluster domain (Zn_2_Cys_6_), C_2_H_2_ zinc finger domain, GATA zinc finger domain, HTH domain, bZIP (basic leucine zipper) domain, and bHLH (basic helix–loop–helix) domain (Figure 1). Each transcription factor recognizes and binds to a specific DNA sequence located in the regulatory region of target genes—such as promoters and enhancers—via its DNA-binding domain, and this sequence is defined as a cis-acting element (examples include the TATA box, GC box, and hormone response element) [5]. The TRD mainly consists of a transactivation domain (TAD) and a repression domain (RD). The transactivation domain encompasses acidic amino acids (acidic activation domain) and glutamine/proline-rich domains [6]. The KRAB domain is a canonical repression domain, which exerts its function by recruiting the core repressor complex KAP-1 (KRAB-associated protein 1). However, no typical KRAB domain has been identified in fungi [7].

Moreover, the dimerization domain is crucial for DNA-binding specificity, regulatory diversity, and stability, enabling transcription factors to form homodimers or heterodimers [8]. Among these, the homologous or heterologous dimers unique to fungi are classified into the zinc cluster dimerization domain (Zn_2_Cys_6_) and the Velvet domain. For example, in fungi such as *Aspergillus nidulans*, there are four core members: VeA (Velvet), VelB (Velvet-like B), VelC (Velvet-like C), and VosA (viability of spores A) [9].

### 1.2. Classification of Transcription Factors

**Figure 2:**
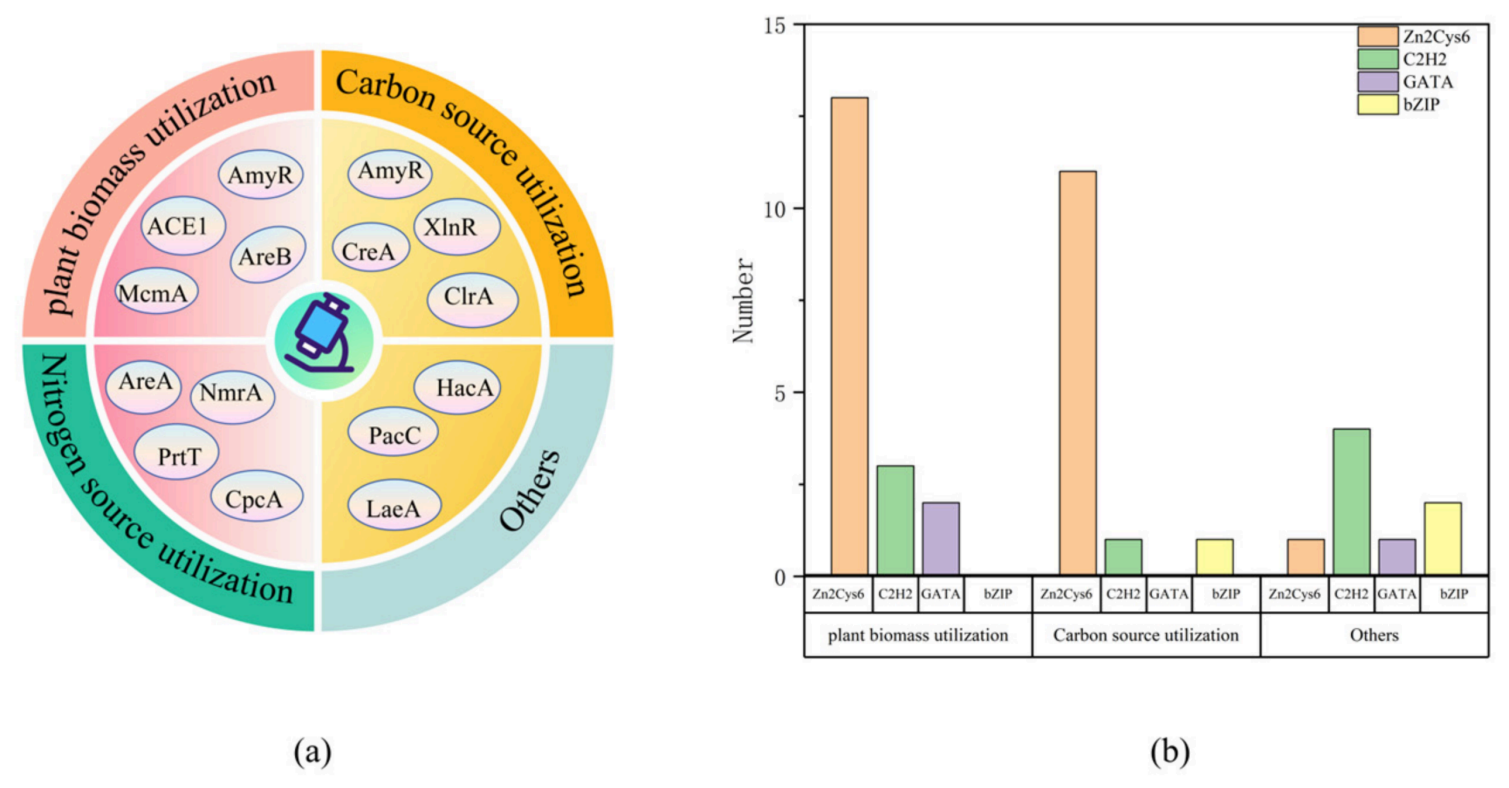
Transcription factor classification and number in different pathways. (**a**) Transcription factor classification in filamentous fungi. (**b**) Statistics of partial transcription factors in fungi. The data were compiled from a systematic survey of published literature.

To date, numerous diverse transcriptional regulatory factors have been reported and classified according to the utilization of plant biomass, carbon source, nitrogen source, etc. (Figure 2a) [10]. For instance, ACE1 and AreB are key components of the regulatory network governing plant biomass degradation, XlnR and CreA play core roles in the regulatory network of carbon-source utilization, and NmrA and CpcA ensure that fungi can survive normally under various nitrogen-source conditions. As illustrated in Figure 2b, among the transcriptional regulators involved in the utilization of plant biomass and carbon sources, the Zn_2_Cys_6_ family accounts for the largest proportion, followed by C_2_H_2_-type transcription factors. In addition, bZIP and GATA transcription factors account for a small proportion. Although bHLH transcription factors appear to account for a relatively small proportion of this specific functional category, they remain an important class of regulatory factors in other biological processes [10]. The dominant role of Zn_2_Cys_6_ transcription factors may reflect their specialized function in regulating the complex metabolic networks required for plant cell wall degradation in filamentous fungi. 

Zn_2_Cys_6_, also known as the zinc binuclear cluster protein, is a specific class of transcription factors unique to fungi. In zinc finger structures [11], Zn^2+^ binds to cysteine and histidine residues within the protein sequence. This family of transcription factors contains a conserved C-X_2_-C-X_6_-C-X_5−12_-C-X_2_-C-X_6−8_-C domain (where C represents cysteine, and X represents any amino acid) [12], and its DNA-interacting structure is shown in Figure 1a. The genome sequencing of filamentous fungi and prediction of protein functions based on the PFAM database revealed that this family represents the largest class of transcription factors [13,14,15]. Current research indicates that this family exists as homodimers; recognizes the C(G/C)GNX(C/A)GG motif; and is involved in a variety of biological processes, including growth and development, secretion of extracellular proteins, utilization of carbon and nitrogen sources, primary/secondary metabolism, and stress resistance [11,16]. In terms of C_2_H_2_ transcription factors, they also belong to the zinc finger family of proteins and bind to DNA in the form of monomers. This family contains a conserved zinc finger domain with the sequence Cys-X_2~4_-Cys-X_3_-Phe-X_5_-Leu-X_2_-His-X_3_-His, and the interaction structure between this domain and DNA is shown in Figure 1b. In filamentous fungi, CreA, PacC, and BrlA are three typical transcription factors belonging to the C_2_H_2_ family, among which CreA acts as a transcriptional repressor for carbon-source metabolism [17]. PacC responds to changes in ambient pH and regulates a series of genes that are expressed under acidic or alkaline conditions [18,19]. The primary function of BrlA is to initiate and regulate the entire process of conidiation [20]. GATA transcription factors are a family of factors unique to eukaryotes; this family specifically binds to DNA via the conserved sequence H(A/T)GATAR(A/G) [21], as shown in Figure 1d. This family is primarily involved in the regulation of nitrogen-source metabolism, with examples including AreA and AreB. Notably, the function of the major isoform AreA can be compensated by AreB [22]. The DNA-binding domain of basic leucine zipper (bZIP) family transcription factors is enriched in basic amino acids, and leucine residues are spaced every six amino acids at the carboxyl terminus, forming the leucine zipper region. Two leucine zipper regions form a leucine zipper via hydrophobic interaction; thus, these TFs mostly bind to DNA in the form of dimers [23], as shown in Figure 1c. The regulatory network of the bZIP transcription factor family in Neurospora crassa indicates that bZIP proteins are capable of inducing sustained regulatory responses to external stresses, including oxidative stress, amino acid deprivation, and heavy metal stress, with CPC-1 and HAC-1 serving as typical examples [24]. The bHLH family is a class of highly conserved transcription factors in eukaryotes. The interaction structure between bHLH transcription factors and DNA is shown in Figure 1e. In plants or yeast, the bHLH domain mainly binds to DNA via the G-box sequence (CACGTG) and can regulate biological processes, including development (e.g., mycelial differentiation and sporulation), metabolism (e.g., iron metabolism), and light response [25,26].

### 1.3. Positive and Negative Regulation of Transcription Factors

Gene expression in fungi is regulated by specific transcription factors. Positive regulatory factors typically activate gene transcription and upregulate gene expression levels by recruiting transcription coactivator complexes (such as histone acetyltransferases), promoting the assembly of RNA polymerase II, or stabilizing the transcription initiation complex [27]. Negative regulators, on the other hand, may suppress gene transcription and downregulate gene expression levels by blocking the binding of positive regulators, recruiting transcriptional co-repressor complexes (such as histone deacetylases), or directly interfering with the regulation of the underlying transcriptional network.

#### 1.3.1. Positive Regulation

**Figure 3:**
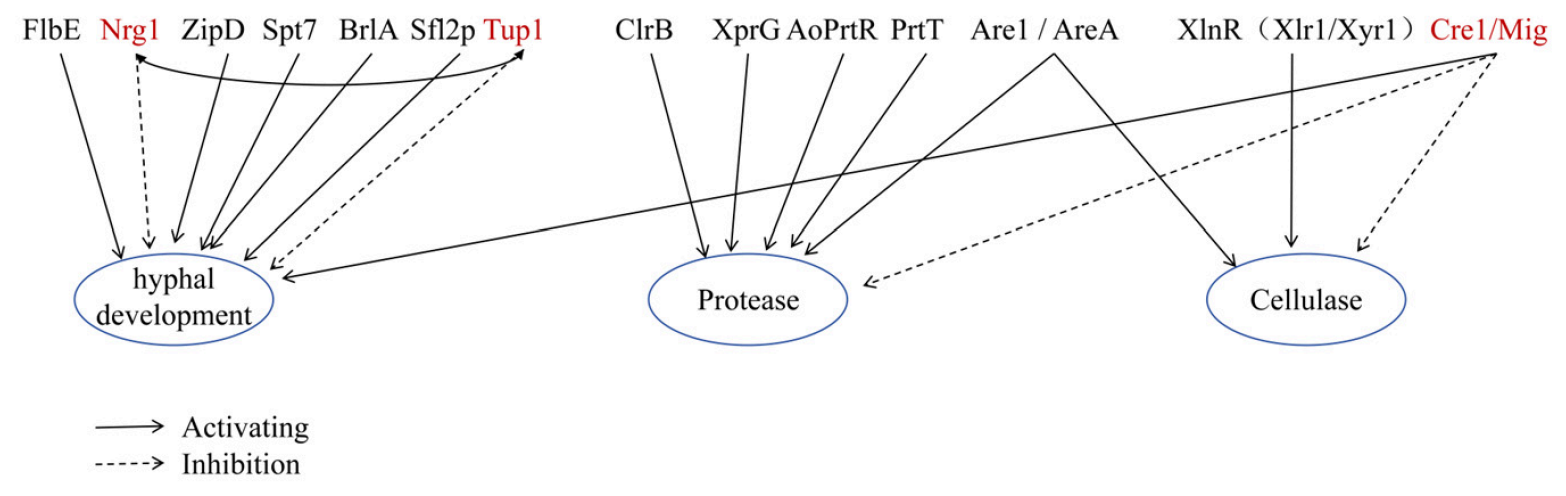
Transcription factors involved in hyphal growth and enzyme metabolism in filamentous fungi. The negative regulators are shown in red.

To date, it has been established that the major transcriptional activation regulators in fungi include Sfl2p, GAL4, XlnR, Ste12, Cmr1p/Pig1p, AcuK/AcuM, and StuA/BrlA (Figure 3). Among these regulators, Sfl2p can positively regulate mycelial development as a promoter of mycelial morphology under elevated temperature conditions (25 °C → 37 °C) [28]. Ste12 affects gene regulation in a light-dependent manner, which is cellulose-dependent. It further participates in the regulation of cellulase gene expression and carbon source-dependent growth by regulating genes encoding various carbohydrate-active enzymes (CAZymes), as well as genes involved in secondary metabolism [29]. Interestingly, some transcription factors contain both C_2_H_2_ and Zn_2_Cys_6_ binuclear cluster, two distinct types of DNA-binding domains; for example, Cmr1p and Pig1p are key transcription factors identified in *Colletotrichum lagenarium* and *Magnaporthe grisea*, respectively. Cmr1p positively regulates melanin biosynthesis in mycelia and does not affect the expression of genes irrelevant to melanin synthesis. Pig1p specifically regulates the expression of melanin synthesis genes during the mycelial stage of *Magnaporthe grisea* and has no significant effect on melanin synthesis during the appressorium stage [30]. *Trichoderma reesei* Xyr1 was initially shown to be a xylan-induced transcriptional regulator involved in the utilization of D-xylose through the pentose catabolic pathway and to specifically recognize the GGC(A/T)_3_ sequence [31]. Studies have shown that multiple Xyr1 recognition sites exist upstream of numerous cellulase and hemicellulase genes. Xyr1 exhibits the strongest binding affinity for the GGCTAA motif and therefore acts as a core transcriptional regulator in the cellulose degradation process [32]. Furthermore, the transcription factor domain of *Trichoderma reesei* Xyr1 is crucial for its downstream regulatory pattern, and a single base mutation, A824V, can relieve carbon catabolite repression on the expression of the Xyr1 transcription factor and cellulases, enabling efficient cellulase production on various carbon sources [33].

#### 1.3.2. Negative Regulation

The common transcriptional repressor regulators in fungi mainly include Sfl1p, Tup1, Mig1, GAL80, and VosA. Tup1 is a highly conserved global transcriptional co-repressor in fungi (Figure 3). It typically forms a complex with the Cyc8/Ssn6 protein to synergistically repress the expression of a large number of genes, thereby regulating various biological processes, including growth, development, and stress response [34]. Studies have shown that, in *T. reesei*, Cre1 transcription is activated in the presence of readily available carbon sources such as glucose. Cre1 specifically binds to the SYGGRG motif on the promoter and regulates the transcription of downstream genes: 47.3% of these genes, such as cellulase, are repressed by Cre1, while 29.0% are activated by Cre1 [35]. For example, the high-yield cellulase strain Rut-C30 was derived from the parental strain QM6a through mutagenesis; analysis revealed that Cre1 in Rut-C30 had undergone partial truncation. Although this truncated Cre1 retains DNA-binding activity, it is unable to effectively repress the cellulase gene, thereby allowing the Rut-C30 strain to partially alleviate the carbon product repression (CCR) effect [36].

### 1.4. The Current Bottleneck Faced by Research

Although the central role of transcription factors in the growth, development, metabolism, and stress responses of filamentous fungi is widely recognized, and genomics and functional genomics technologies have greatly expanded our understanding of the transcription factor landscape, this field still faces significant challenges. Firstly, there is functional overlap and complex cross-regulation among transcription factors, which means that single-gene knockout does not produce significant effects, thereby making it difficult to influence the entire regulatory network. For example, FfmA and AtrR share more than 300 common targets; they interact with each other and regulate each other’s expression. If one is knocked out, the other compensates, so the results of a single-gene knockout are limited and fail to reflect the network of synergistic regulation [37]. Secondly, the editing efficiency of multi-gene editing decreases as the number of target genes increases. For example, a study found that, in *Aspergillus oryzae,* the editing efficiency of single-gene editing was 37.6%, while that of double-gene editing was 19.8% [38].

## 2. *Aspergillus* sp.

The genus *Aspergillus* comprises approximately 260 to 837 species, with common species including *Aspergillus fumigatus*, *Aspergillus nidulans*, *Aspergillus flavus*, and *Aspergillus niger*. Currently, *Aspergillus* is widely employed in industry for the production of a variety of organic acids, including citric acid, gluconic acid, and L-malic acid, and multiple types of extracellular enzymes, including β-galactosidase, β-mannosidase, xylanase, and amylase [39].

### 2.1. Homologous Transcriptional Regulatory Factor

There is a class of transcriptional regulatory factors that exhibit high homology across different species, although the specific characteristics of their regulatory networks may undergo subtle changes due to environmental adaptation. This class of factors regulates the basic biological activities of *Aspergillus*, including, but not limited to, growth and development, sporulation, carbon- and nitrogen-source metabolism, and stress responses.

#### 2.1.1. Strain Development and Sporulation

**Figure 4:**
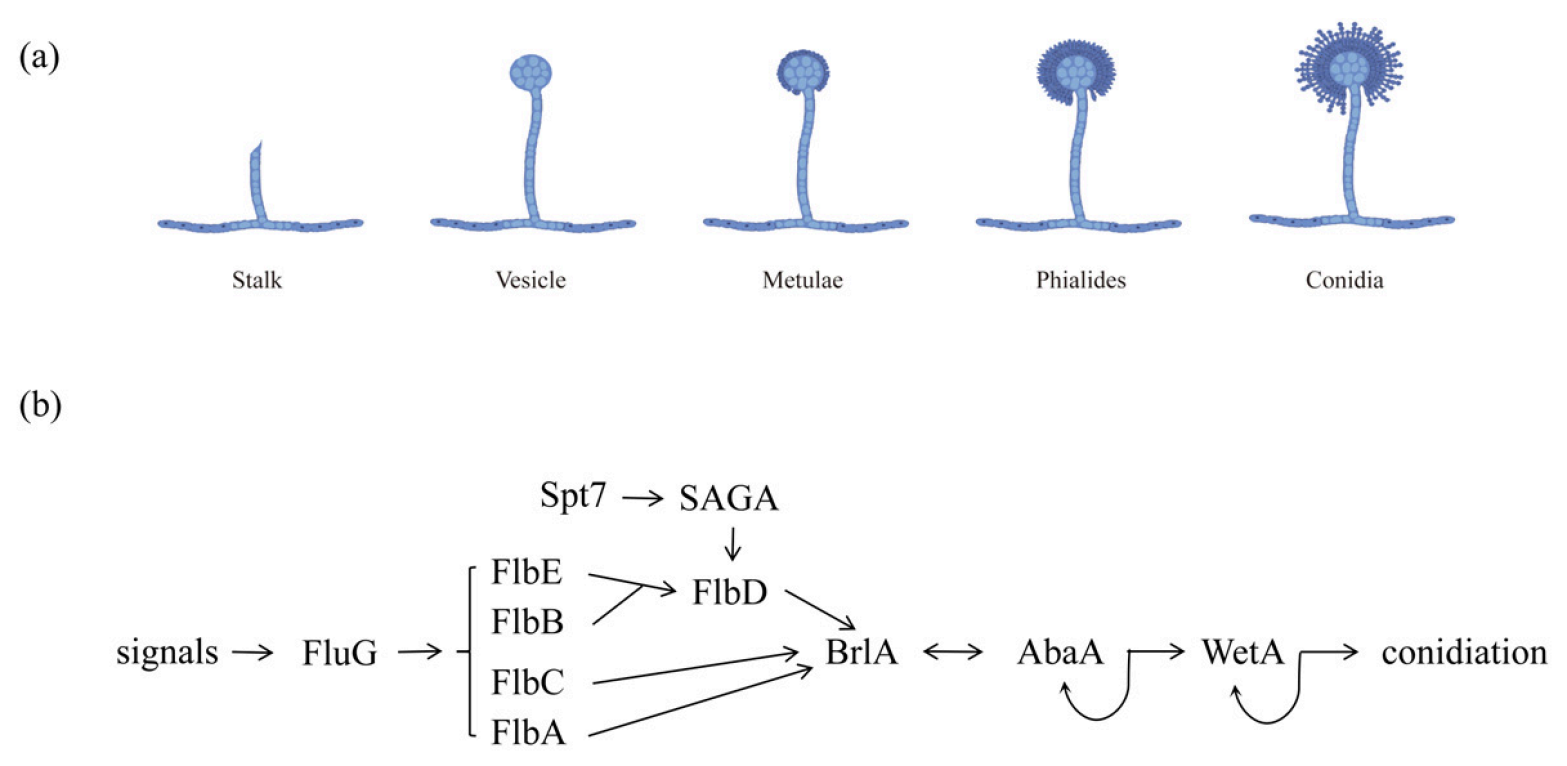
The conidia development and regulatory network of *Aspergillus*. (**a**) The developmental process of *Aspergillus* conidia. (**b**) *Aspergillus* conidial development and transcription factor regulatory network.

In *Aspergillus*, the development and maturation of aerial conidia and conidia are governed by the Central Developmental Pathway (CDP), which drives the formation of hyphae and chained conidia (Figure 4). In *Aspergillus nidulans*, the CDP is activated by BrlA to initiate conidiophore development and subsequently activates AbaA and WetA sequentially during the middle-to-late stages of conidiophore development. The sequential activation of BrlA–AbaA–WetA not only transcriptionally activates downstream genes involved in the initiation of conidiation but also promotes the biosynthesis of spore wall components [20]. BrlA is currently the most thoroughly characterized transcription factor that regulates conidiation during asexual reproduction in *Aspergillus*. BrlA is localized at the most upstream position of the central pathway governing conidiophore formation, and it induces the expression of multiple asexual reproduction regulators, including AbaA and WetA downstream [40]. For the activating factor AbaA, its encoded product is involved in the core regulatory pathway of feedback repression, which activates downstream regulatory genes, including WetA and VosA, as well as structural genes, including *yA* and *chsA*, and accelerates the process of spore development. AbaA is regulated by BrlA, and, in turn, it can enhance the expression of structural genes induced by BrlA. Therefore, the two act as mutual transcriptional activators [41]. As the third transcription factor in the central regulatory pathway, WetA, similar to the first two genes, acts at the late stage of the conidiation pathway and is involved in the biosynthesis of key cell wall components. As shown in Figure 4b, a recent study revealed that Spt7 can globally regulate the transcription of sporulation-related genes by maintaining the function of the SAGA complex and consequently upregulates the expression of FlbD and BrlA [42].

When vegetative hyphae differentiate and specialize to form conidiophores, multiple genes are required to participate before the activation of the *brlA* gene. Among these, genes that exert positive regulation in a non-linear pattern on the central regulatory pathway are defined as upstream developmental activation transcription factors (UDAs), also known as the fluffy locus. All UDA mutant strains are incapable of conidiospore production and exhibit low expression levels of the *brlA* gene [43], with most colonies displaying a white villous morphology. Classification of the corresponding genes based on their BrlA-inducing capability yielded a primary grouping including FluG and FluA–FluE.

#### 2.1.2. Metabolism of Carbon and Nitrogen Sources

When multiple carbon sources are simultaneously available to microorganisms, they preferentially utilize the carbon source with the highest energy production efficiency (typically glucose) while simultaneously suppressing the synthesis of enzymes and transporters associated with the catabolism of other, less efficient carbon sources—such as lactose, galactose, and xylose—at the transcriptional, translational, and transport levels. This global gene regulatory mechanism, which enables the hierarchical utilization of carbon sources, is known as carbon product-mediated repression (CCR) [44]. In the filamentous fungus *Aspergillus*, the core regulator of carbon product-mediated inhibition, CreA is a C_2_H_2_-type transcription factor that regulates the transcription of target genes by binding to a regulatory motif (5′-SYGGRG-3′) within their promoters [45]. In *A. niger*, CreA regulates the expression of cellulases and hemicellulases by binding to their promoters or by suppressing the expression of transcriptional activators.

Nitrogen metabolism regulation is a multilayered system consisting of three aspects: catabolism of nitrogen sources; anabolism of amino acids; and protease regulation, involving transcription factors AreA, AreB, NmrA, CpcA, and PrtT. Its core regulatory mechanism is nitrogen catabolite repression. When sufficient preferentially utilizable nitrogen sources (such as ammonium salts and glutamine) are present in the environment, fungi will repress the expression of related genes that catabolize other non-preferential nitrogen sources (such as nitrates and certain amino acids) to achieve efficient energy utilization, and the core of this regulatory network is the GATA transcription factor AreA and its co-repressor NmrA [46]. AreB is an additional GATA transcription factor that forms an interaction network with AreA to co-regulate nitrogen-source metabolism [22]. Under conditions of amino acid starvation induced by nitrogen/oxidative stress, CpcA initiates emergency regulation to upregulate global amino acid biosynthesis. As the primary sensory gene for nitrogen-source supply, PrtT regulates proteases to acquire nitrogen from proteins.

#### 2.1.3. Stress Response Factor

A class of stress response factors exists in fungi, which have consistently played a core role in enabling strains to adapt to environmental changes during the evolutionary process. Protein sequences, functions, and structures are highly similar across different species and, thus, are characterized by strong evolutionary conservation [47]. For example, the transcription factor PacC (from *A. nidulans*)/Rim101 (from *Saccharomyces cerevisiae*) regulates the core pH regulatory network. In previous studies, the generation of PacC-deficient mutants in *A. niger* confirmed that PacC plays a central role in regulating the expression of acid phosphatase [48]. Even when a particular stress response regulator acts as the central regulator of a specific function, the target genes activated may vary among strains under different growth conditions. For example, the acidophilic *A. niger* and neutrophilic *A. fumigatus* exhibit completely distinct repertoires of PacC-activated target genes, which respectively express enzymes and transporters required for their optimal pH environments [49].

In addition to PacC/Rim101, other regulatory factors essential for survival are also highly conserved, including the heat shock factor HsfA and the bZIP-type transcription factor AtfA [50,51].

### 2.2. Non-Homologous Transcription Regulators

#### 2.2.1. Specific Regulators of Secondary Metabolites

Secondary metabolites exhibit the most significant divergence and are the most species-specific. The biosynthetic gene clusters of each secondary metabolite, including toxins, pigments, and antibiotics, are typically regulated by cluster-specific transcription factors.

LaeA is a global regulatory factor first identified in *A. nidulans*, and it is also a conserved global secondary metabolism regulatory factor in filamentous fungi. LaeA contains a critical S-adenosylmethionine (SAM)-dependent methyltransferase domain (MTD) [3,52]. A distinctive feature of LaeA is that it does not bind to DNA directly [53,54] but regulates gene expression through methylated histones. LaeA plays a critical role in *A. niger*: it is not only essential for high-yield citric acid production but also acts as a positive regulator of toxin biosynthesis while supporting normal growth and stress tolerance of the strain [3,52]. Meanwhile, in *A. flavus*, LaeA regulates cellular morphology and promotes the production of secondary metabolites (SMs), including cyclopiazonic acid and aflatoxin [55]. In addition, VeA is a global regulatory protein that is highly conserved in filamentous fungi. Different from LaeA, a methyltransferase, VeA is capable of directly binding to DNA. Its most prominent feature is that its function is precisely regulated by light, and it acts as a core hub linking environmental signals to regulate fungal development and metabolism [4].

Unlike LaeA, transcription factors such as KojR and AflR are not methyltransferases that exert functions via epigenetic modifications but belong to canonical transcription factors that directly bind to DNA. In *A. flavus*, the AflR protein binds to at least 17 genes within the aflatoxin biosynthetic gene cluster, activating enzymatic cascade reactions to enable the biosynthesis of aflatoxin [56,57]. The promoter region of specific biosynthetic genes is bound by AflR in the form of a dimer, which has the 5′-TCG(N_5_)CGA-3′ binding motif (Table 1) [58]. AflR also recognizes the binding sequences 5′-TTAGGCCTAA-3′ and 5′-TCGCCCGG-3′ [59]. Furthermore, in the parasitic *A. parasiticus*, the AFLR transcription factor binding sites of the *nadA*, *hlyC*, and *niiA* genes were identified outside the aflatoxin gene cluster, providing the first evidence that genes outside the aflatoxin gene cluster may be regulated by AflR [60]. In *A. oryzae*, KojR is a typical Zn(II)_2_Cys_6_ zinc cluster protein that functions as a specific activator of the kojic acid biosynthesis pathway and exerts regulatory effects [61]. In *A. fumigatus*, SrbA regulates sterol metabolism and hypoxic response, which is critical for its survival in the hypoxic environment of the host lung [62].

#### 2.2.2. The Regulatory Ability to Decompose Substrates

Although both XlnR and AmyR in *Aspergillus* regulate cellulose/hemicellulose depolymerization and starch decomposition, their regulatory intensity, target genes range, and induction conditions may differ.

The regulatory networks of XlnR and AmyR in *A. niger* are likely more extensive and efficient, enabling them to synergistically activate a large number of genes encoding auxiliary enzymes and transporter proteins, which matches its role as an excellent decomposer of plant polysaccharides. As a Zn(II)_2_Cys_6_-type transcription factor, AmyR can regulate the expression of amylolytic enzymes in *Aspergillus* fungi [63,64]. This protein regulates the transcription of genes encoding major starch-degrading enzymes by binding to the direct repeat sequence of CGG triplets (with an 8-base pair interval) in the amylase gene promoter region, or the CGGN_8_AGG sequence within the amylase gene promoter [65,66]. When the expression of starch decomposition genes is induced by maltose and isomaltose generated from starch degradation, AmyR is promoted to rapidly translocate from the cytoplasm to the nucleus, thereby activating the transcription of amylase genes [67]. However, glucose, another starch degradation product, triggers carbon catabolite repression, thereby inhibiting the expression of starch decomposition-related genes [68]. It is worth noting that, in *Aspergillus oryzae* and *Aspergillus niger*, it has been demonstrated that glucose can also induce the production of α-amylase through an AmyR-dependent mechanism [69].

XlnR is another Cys_6_-type zinc finger transcription factor, which was initially identified in *A. niger*. In addition to regulating auxiliary enzymes related to xylan degradation, it is also involved in the transcriptional regulation of genes encoding major xylanases and cellulases [70]. XlnR directly binds to the GGCT(A/G)A sequence in the promoters of various xylanase-encoding genes. Its DNA-binding domain is located at the N-terminus, and multiple motifs exist at the C-terminus, among which the coiled-coil region is involved in the nuclear import of XlnR [71,72]. Another C-terminal motif downstream of the coiled-coil region harbors a glucose repression motif and may be involved in the regulation of XlnR activity [70,73]. Recent studies have demonstrated that XlnR from *A. oryzae* is highly phosphorylated in the D-xylose-induced transactivation state, indicating that D-xylose is an effective inducer of XlnR-dependent genes [74]. Furthermore, this protein is highly abundant in acid-pretreated straw hydrolysate. Therefore, using acid-pretreated straw hydrolysate as a carbon source is a promising approach to induce the expression of XlnR-dependent genes [69]. Furthermore, Delmas et al. confirmed that *A. niger* secretes a variety of plant cell wall-degrading enzymes when wheat straw is used as the carbon source, including enzymes encoded by XlnR-dependent genes [75,76]. For *A. oryzae*, its regulatory network may be more biased toward the efficient conversion of starch and protein, which is consistent with its application in traditional fermentation brewing.

## 3. *Trichoderma* sp.

### 3.1. Trichoderma reesei Regulatory Factor

**Figure 5:**
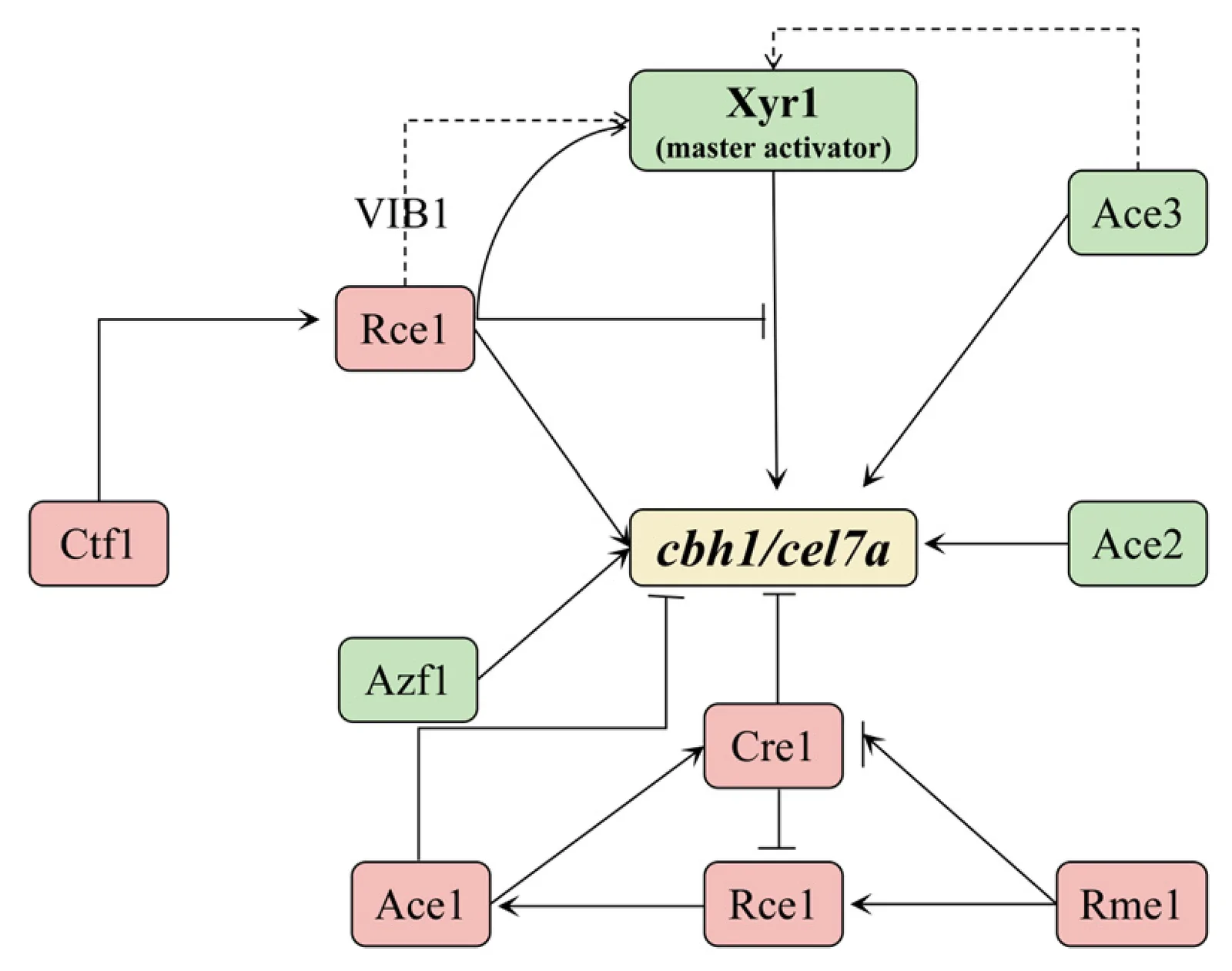
The key transcription factor for *Cel7a* secretion in *T. reesei*. The diagram illustrates the multilayered transcriptional regulation of the major cellobiohydrolase-encoding gene *Cbh1*/*Cel7a* (highlighted in the center). Positive and negative regulators are represented by green and red boxes, respectively. Arrows (→) indicate transcriptional activation, whereas perpendicular lines (⊢) denote transcriptional repression. Solid lines represent direct DNA binding and transcriptional regulation at the promoter level, while dashed lines indicate indirect regulation.

*T. reesei* can produce cellulase at a high yield under cellulose induction (Figure 5). The main components include the major cellobiohydrolases *Cbh1*/*Cel7a* and *Cbh2*/*Cel6a*, the endoglucanases *Egl1*/*Cel7b* and *Egl2*/*Cel5a*, and the β-glucosidase *Bgl1*/*Cel3a*. These cellulases account for 90% of the extracellular secreted enzyme system of *T. reesei*, among which cellobiohydrolase *Cbh1*/*Cel7a* accounts for more than 60% of the total cellulase content [77]. In recent years, key regulatory factors involved in the cellulose induction process and transcription factors modulating cellulase expression in *T. reesei* have been gradually elucidated. Xyr1 has also been verified as the most critical transcriptional activator that regulates the expression of cellulases and hemicellulases. The expression of cellulase-encoding genes is regulated at multiple levels, including the sensing of inducing signals, signal transduction, and transcriptional regulation. Transcription factors including Xyr1, Ace1, Cre1, and Vib1 regulate the expression of cellulase-encoding genes by binding to the promoter regions of these genes.

#### 3.1.1. *T. reesei* Positive Regulatory Factor

Genes associated with cellulose and hemicellulose degradation are regulated by the binuclear zinc cluster protein Xyr1, which serves as the primary transcriptional activator for the expression of holocellulases. Even in the absence of inducing factors, the overexpression of Xyr1 still promotes the production of cellulase [78]. Ace3, as another important transcriptional activator of cellulase expression, directly mediates the expression of cellulases by binding to the promoter of cellulose-degrading genes [79].

The transcription factor Azf1 is homologous to Azf1p in *S. cerevisiae*. Azf1 does not regulate the cell cycle of *T. reesei*, but it directly regulates the expression of cellulase-encoding genes by binding to the promoters of *cel7a*, *cel45a*, and *swo* genes. Deletion of Azf1 reduces the expression of cellulose and hemicellulose decomposition-related genes [80].

This study reveals that the expression of cellulase and protease is coupled under the regulation of the GATA-type transcription factor Are1. However, the regulatory mechanisms governing cellulase and protease production exhibit significant differences. When the nitrogen source is sufficient, Are1 can activate the expression of cellulase and inhibit the expression of protease. When nitrogen sources are insufficient, the expression of proteinase is activated, while that of cellulases is suppressed [81]. Taken together, these results indicate that Are1 most likely acts as a mediator regulating the expression of cellulases and proteases, and promotes the balanced utilization of carbon and nitrogen sources.

#### 3.1.2. *T. reesei* Negative Regulator

Carbon catabolite repression (CCR) is a global regulatory system that is present in almost all heterotrophic hosts and is mainly regulated by cellulase transcriptional repressor Cre1 and activators including Xyr1, Clr1, and Clr2 (Table 2). It is worth noting that the accumulation of monosaccharides generated during cellulose degradation, such as D-glucose, triggers carbon catabolite repression (CCR) and significantly downregulates the transcription of cellulose-degrading enzyme genes in *T. reesei* [82]. Endogenous Cre1, a C_2_H_2_-type zinc finger protein, can bind to the conserved sequence in the promoter region of target genes, thereby inhibiting the transcription of cellulase-encoding genes [83].

Other transcriptional repressors similar to Cre1 include Ctf1, Rme1, and RCE1. A study found that, when the strong promoter *pdc1* was selected to overexpress *ctf1* in *T. reesei* RUT-C30, cellulase production was significantly reduced. Conversely, the deletion of *ctf1* upregulates the transcription of VIB1 while downregulating the transcription of Rce1, which further upregulates the transcription of XYR1 and ACE3, ultimately activating the transcription of cellulase genes [84]. Rme1 inhibits cellulase expression by directly suppressing the expression of the major cellulase Cel7a and activating the expression of the primary inhibitor Cre1 [85].

**Table 2:** Transcription factors involved in cellulase regulation in *T. reesei*.

Name	Class	Functions	Motif	Reference
Xyr1	Zn(II)_2_Cys_6_	Activate cellulase gene	GGC(A/T)_3_	[86]
Ace2	C_2_H_2_	Regulation of cell separation	GGCTAATAA	[87]
Ace3	Zn(II)_2_Cys_6_	Activate cellulase gene	CGGAN(T/A)_3_	[79]
Ace4	Zn(II)_2_Cys_6_	Activate cellulase gene	two adjacent -GGCC-	[88]
BglR	Zn(II)_2_Cys_6_	Regulating β-glucosidase gene	-	[89]
Are1	GATA	Regulator of nitrogen metabolism	HGATAR	[81]
Rxe1	C_2_H_2_	Activate cellulase gene	-	[90]
Azf1	C_2_H_2_	Regulator of nitrogen metabolism	-	[91]
Crz1	C_2_H_2_	Respond to calcium ion signals	(T/G)GGCG or GGGC(G/T)	[92]
Pac1	C_2_H_2_	Regulate pH gene	GCCARGb	[93]
Cre1	C_2_H_2_	Mediating carbon metabolism repression	SYGGRG	[35]
Ypr1	Zn(II)_2_Cys_6_	Regulate the synthesis of secondary metabolites	-	[94]
Rce1	C_2_H_2_	Inhibit cellulase gene	GGC(A/T)_3_	[95]
Rce2	C_2_H_2_	Inhibit cellulase gene	(T/A)NNNNCCG	[96]
Ctf1	C_2_H_2_	Inhibit cellulase gene	-	[92]

### 3.2. Regulatory Mechanisms of Transcription Factors

Most cellulase and hemicellulase genes are coordinately regulated by multiple transcription factors in *T. reesei* [97]. A novel dual-core zinc cluster transcription factor (JGI|Trire2|123881) has been identified that inhibits xylanase activity without exerting an inhibitory effect on cellulase activity, and it is defined as SxlR (xylanase-specific regulator). SxlR binds to the promoters of GH11 xylanase genes (*xyn1*, *xyn2*, and *xyn5*) but does not bind to the promoters of the GH10 xylanase gene (*xyn3*) or GH30 xylanase gene (*xyn4*), which indicates that fungi do not need to produce all xylanases simultaneously. Instead, they only need to produce specific enzymes selectively to avoid resource waste [98].

ACE3 is essential for lactose metabolism, primarily mediating this biological process via the regulation of CRT1 and BGA1. ACE3 can bind to the promoters of most cellulase-encoding genes, thereby directly mediating cellulase production. Additionally, it can bind to *crt1* and *xyr1* to indirectly regulate cellulase production [79].

Previous studies have elucidated the L-fucose response system in the model filamentous fungus *T. reesei*, which mediates plant cell wall degradation. The core of this system is the transcription factor Fur1, which is indispensable for the biosynthesis of L-fucose. FUR1 mediates the expression of the majority of enzymes triggered by L-fucose, which target various glycosidic bonds in complex carbohydrates (e.g., β-glucuronide, α-galactoside, and β-xyloside bonds) [99].

## 4. *Penicillium* sp.

Among the current studies on transcriptional regulatory factors in *Penicillium* fungi, the most extensively investigated are those involved in the regulation of the expression of plant cell wall-degrading enzymes, such as cellulases and hemicellulases. In *A. niger*, *A. fumigatus*, and *Penicillium oxalicum*, ClrB acts as the primary transcriptional regulator responsible for activating genes encoding cellulases; the absence of this regulator results in a significant reduction in cellulase production in the strains. However, current studies have shown that, in *Penicillium verruculosum*, XlnR and ClrB have different functional effects on the expression of key cellulase-encoding genes: XlnR exerts a significant regulatory effect on *cbh1*, ClrB exerts a moderate regulatory effect on *egl2*, and neither of these transcription factors has a noticeable effect on *bgl1* [100]. In *P. subrubescens*, AraA has a more significant effect on plant biomass synthesis than its homolog XlnR [101].

The same regulatory factor also exhibits functional differences across *Penicillium* species (Table 3). For example, VeA, one of the global regulatory factors, was first identified in *A. nidulans*, and its primary function is to regulate growth, development, and secondary metabolism. In *P. chrysogenum*, PcVeA, the homolog of VeA, acts as a positive regulator of penicillin biosynthesis, and deletion of the PcVeA gene leads to abnormal hyphal branching [102]. In *P. expansum*, deletion of the VeA gene blocks the production of patulin and citrinin, while in Penicillium citrinum, the VeA gene positively regulates the biosynthesis of mevastatin, a cholesterol-lowering drug, by controlling the expression of the cluster-specific activator MlcR [103]. Second, another global transcriptional regulator, LaeA, exhibits a broader regulatory spectrum in *P. expansum* and positively regulates the biosynthesis of multiple secondary metabolites, including patulin and roquefortine C. However, in *P. oxalicum*, it does not affect the biosynthetic gene cluster of roquefortine C [104]. In addition, the transcription factor CreA that mediates CCR directly regulates penicillin synthesis in *P. chrysogenum*, while in *P. expansum*, CreA not only regulates metabolism but also acts as a key virulence factor [105].

## 5. Genetic Engineering Modification Strategy

In filamentous fungi, multilevel transcription factor regulatory networks influence the expression of hemicellulose, cellulose, and secondary metabolites. These include global regulatory factors such as CreA/Cre1 and LaeA/VeA to pathway-specific activators such as Xyr1, Ace3, AmyR, AflR, and KojR, as well as repressors such as Rce1, Rce2, and Ctf1. Studies on the functions of these transcription factors in the genera *Aspergillus, Trichoderma*, and *Penicillium* have laid the foundation for the genetic engineering of these strains.

First, we discuss the overexpression and knockout of transcription factors. The expression of genes encoding cellulases and hemicellulases in *T. reesei* is tightly regulated at the transcriptional level. Accordingly, deletion and/or overexpression of transcription factors (TFs) that specifically regulate the expression of xylanase-encoding genes represents a straightforward approach for knowledge-driven strain design [98]. A study found that the overexpression of XYR1 mediated by the tcu1 promoter not only overcame the inhibition caused by cellulase-derived carbon byproducts but also enabled its full expression on non-inducing carbon sources [78]. In *Penicillium* strains used for penicillin production, a study utilized the gpdA constitutive promoter to overexpress LaeA, resulting in a 20–40% increase in penicillin yield while simultaneously activating the synthesis of certain previously silenced secondary metabolites [52]. Conversely, deletion of the natural repressor Cre1—a C_2_H_2_-type transcription factor that mediates the suppression of carbon metabolism by binding to the 5′-SYGGRG-3′ motif—has been shown to effectively lift glucose-induced suppression.

Next, we discuss the construction of chimeric transcription factors. Fungal transcription factors consist of distinct DNA-binding domains (DBDs) and transcription-activating domains (TADs), which makes it possible to construct chimeric transcription factors with customized regulatory properties. Previous reports have confirmed that a novel chimeric transcription factor, composed of the DNA-binding domain of Xyr1 and the transcriptional activation domain of either Ypr1 or Ypr2—both of which are regulatory factors of the sorbitol biosynthesis gene cluster—can effectively perform its function [106]. The fusion of Xyr1 and Ypr2 produces a transcription factor with moderate transcriptional activation activity, whereas the fusion of Xyr1 and Ypr1 produces a transcription factor with very high transcriptional activation activity; moreover, the latter’s inductive activity for xylanase and cellulase is virtually unaffected by the carbon source. Notably, high-level xylanase production has been achieved on glycerol-based medium [107]. Furthermore, researchers have achieved constitutive expression of cellulase under non-induced conditions by constructing a chimeric transcription factor that fuses the DNA-binding domain of ClrB with the regulatory and activation domains of XlnR [108].

To systematically analyze the roles of SdrA, WarA, and WarB in tolerance to weak acids, this study generated single-, double-, and triple-gene knockout strains. Phenotypic analysis revealed that the responses of these three genes to sorbic acid stress exhibited a significant additive effect [109].

Other studies have implemented dual modifications to transcription factors: the combination of TerR overexpression and StuA knockout exerts a synergistic effect on the transcriptional activation of the terrein biosynthetic gene cluster, which increases terrein yield by 25-fold to 12.6 mg/mL with the complete elimination of by-products. This study simultaneously modifies pathway-specific regulators and global transcription factors, which can serve as a general strategy for improving the yield of secondary metabolites produced by other fungi [110].

## 6. Conclusions

Current research has shown that transcription factors are inextricably linked to epigenetic modifications, chromatin complexes, and signal transduction pathways. Research on fungal transcription factors has shifted from studying individual regulatory mechanisms to investigating multifactorial cooperative regulatory networks. For example, cell differentiation is coordinated by developmental regulatory factors and the SWI/SNF chromatin remodeling complex; at the same time, transcription factors also work in concert with enzymes involved in modifications such as histone methylation and acetylation to regulate the expression of metabolic gene clusters. Given the complexity of multilevel regulatory networks involving transcription factors, and since most studies have been limited primarily to model fungal strains such as *Aspergillus*, *Trichoderma*, and *Penicillium*, the specific functions of most predicted transcription factors remain unverified, and the gene cluster silencing specific to fungi requires further investigation.

The research bottlenecks in filamentous fungal transcription factor technology, which stem from their inherent biological complexity and are evident throughout the entire process from basic research to industrial applications, can be addressed by developing more efficient editing tools. For example, the emergence of multi-gene editing systems has effectively overcome the limitations of single-gene editing by enabling the synergistic regulation of activators and transcription factors, the integration of multiple factors, and CRISPR-mediated base editing. The key to overcoming industrial bottlenecks may lie in the development of multi-sgRNA arrays (such as tRNA-sgRNA arrays) and CRISPR base editing. Compared to the insertions or deletions at unintended sites caused by CRISPR/Cas9 editing, base editors—advanced versions of CRISPR editing tools—can induce precise point mutations without causing DNA strand breaks. In the future, label-free base editing systems capable of delivering multiple gRNAs should be developed to enable simultaneous saturation mutations at key sites in multiple transcription factor genes, thereby facilitating a systematic analysis of the functional differentiation of homologous transcription factors.

## Figures and Tables

**Table 1:** Functions of *Aspergillus* transcription factors and their regulatory motifs.

Name	Class	Function	Motif	Strain
SrbA	bHLH	Regulate sterol Metabolism, etc.	TCACGTGGC	*A. fumigatus*
AflR	Zn(II)_2_Cys_6_	Synthesize aflatoxin	TCG (N_5_) CGA	*A*. *flavus*
HapX	bZIP	Sustain iron homeostasis	5′-CSAAT-3′	*A. fumigatus*
ZafA	C_2_H_2_	Regulation of zinc homeostasis	CAA(G)GGT	*A. fumigatus*
KojR	Zn(II)_2_Cys_6_	Kojic acid synthesis	CGGCTAATGCGG′	*A*. *oryzae*
LaeA	-	Regulation of gene expression	-	*A. nidulans*
VeA	Velvet	Growth and development of fungi	-	*A. nidulans*

**Table 3:** Structural classification and functional conservation of key regulatory factors in filamentous fungi.

Name	Class	*Aspergillus* sp. and Functions	*Trichoderma* sp. and Functions	*Penicillium* sp. and Functions	Conservatism
LaeA	Methyltransferase	Global Upregulators of Secondary Metabolism	Regulation of Secondary Metabolism and Enzyme Expression	Regulation of Various Secondary Metabolites	Conservative in function, with differences in the target spectrum
VeA/Vel1	Velvet	Coordination of Development and Secondary Metabolism	Vel1 Regulates Development and Cellulase Activity	Regulation of Secondary Metabolism, Such as Penicillin	Conservative functional framework
CreA/Cre1	C_2_H_2_	Core Inhibitors of Carbon Catabolism	Core Inhibitors of Carbon Catabolism	Regulation of Metabolism and Virulence	Core functions are conservative, while Penicillium species also possess virulence functions
XlnR/Xyr1	Zn_2_Cys_6_	Xylanase/Cellulase Activation	Primary Activators of Cellulase/Xylanase	Regulation of the Cellulase Gene	Functionally conservative, with regulatory intensity varying by species
AmyR	Zn_2_Cys_6_	Amylase Gene Activation	Regulation of the Amylase Gene	Regulation of the Amylase Gene	Limited functionality
PacC	C_2_H_2_	pH-Responsive Core	pH-Responsive Regulation	pH Response and Virulence	Highly conservative
AreA/Are1	GATA	Core of Nitrogen Metabolism	Nitrogen Metabolism and the Protease/Cellulase Balance	Regulation of Nitrogen Metabolism	Limited functionality

## Data Availability

No new data were created or analyzed in this study. Data sharing is not applicable to this article.
